# Supplementary material for: Modified Curcuminoid-Rich Extract Liposomal CRE-SDInhibits Osteoclastogenesis via the Canonical NF-κB Signaling Pathway
Source: Pharmaceutics. 2023 Aug 30;15(9):2248. doi: 10.3390/pharmaceutics15092248 (PMC10537735; doi:10.3390/pharmaceutics15092248)
Supplement: Supplementary file 1 [file pharmaceutics-15-02248-s001.zip › pharmaceutics-2550196-supplementary.pdf]

Supplementary materials

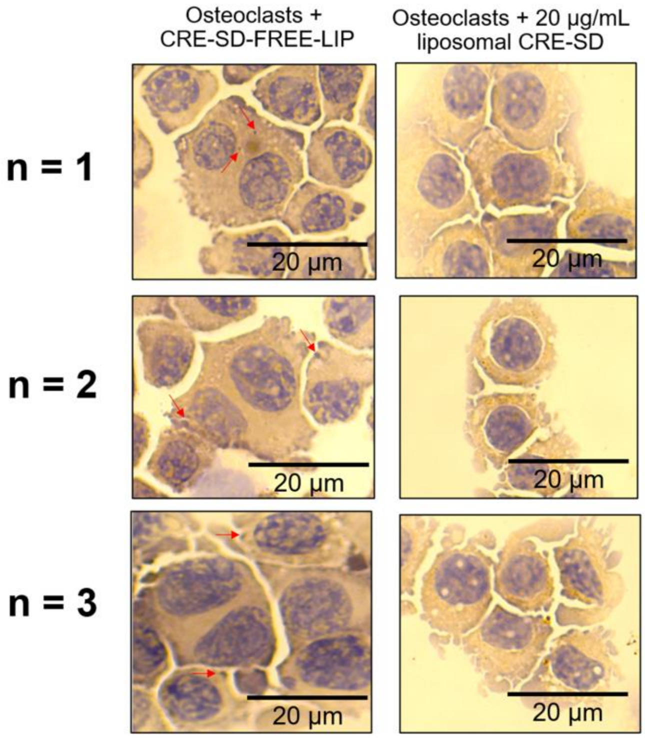

Figure S1. Triplicate microscopy images of 20 ng/mL RANKL-stimulated RAW 264.7 cells (osteoclasts) after TRAP staining, following treatment with CRE-SD-free POPC liposomes (CRE-SD-FREE-LIP) and 20 µg/mL liposomal CRE-SD. The arrows indicate stained granules.

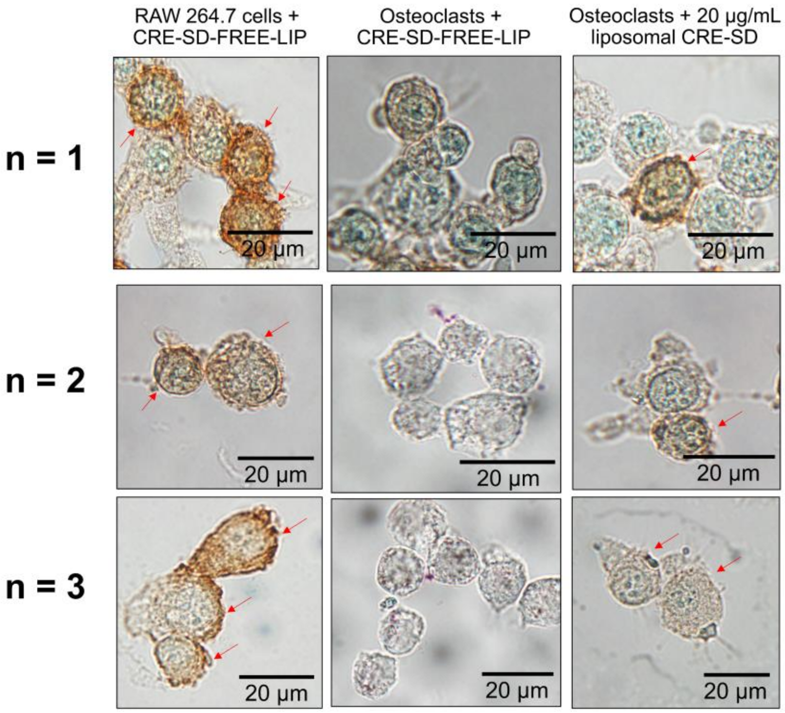

Figure S2. Triplicate microscopy images of 20 ng/mL RANKL-stimulated RAW 264.7 cells (osteoclasts) after the immunocytochemistry analysis of F4/80. RANKL-unstimulated RAW 264.7 cells treated with CRE-SD-FREE-LIP and 20 ng/mL RANKL-stimulated RAW 264.7 cells treated with CRE-SD-FREE-LIP and 20 μg/mL liposomal CRE-SD were examined. The arrows indicate F4/80-positively stained cells.

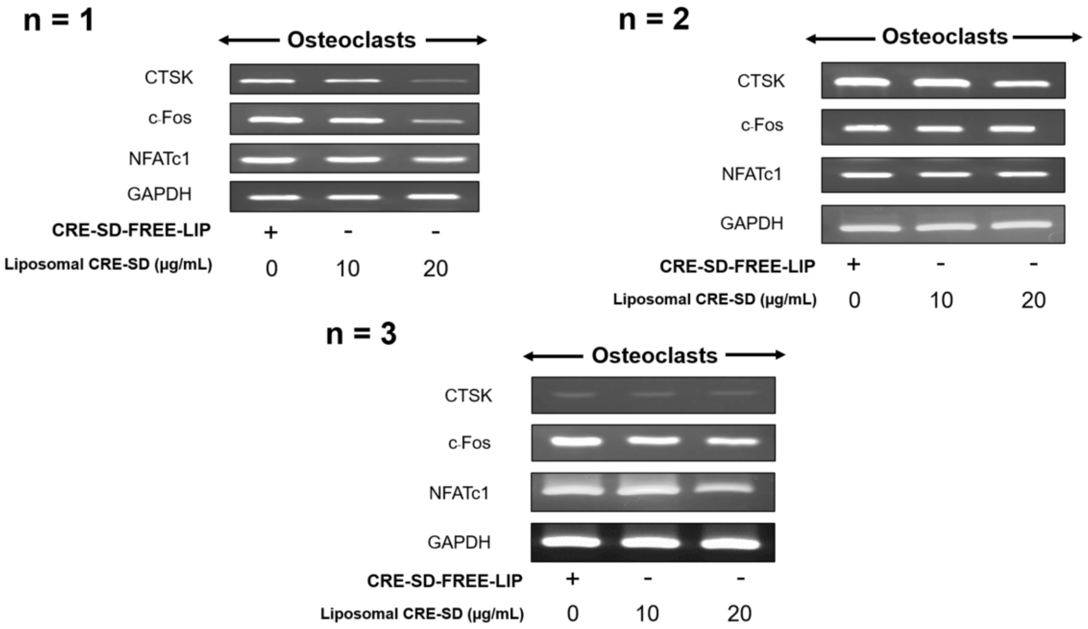

Figure S3. Triplicate images of gels analyzing 20 ng/mL RANKL-stimulated RAW 264.7 cells (osteoclasts) following RT–PCR analysis of CTSK, c-Fos, and NFATc1. The 20 ng/mL RANKL-stimulated RAW 264.7 cells were treated with CRE-SD-FREE-LIP and liposomal CRE-SD (10 and 20 μg/mL).

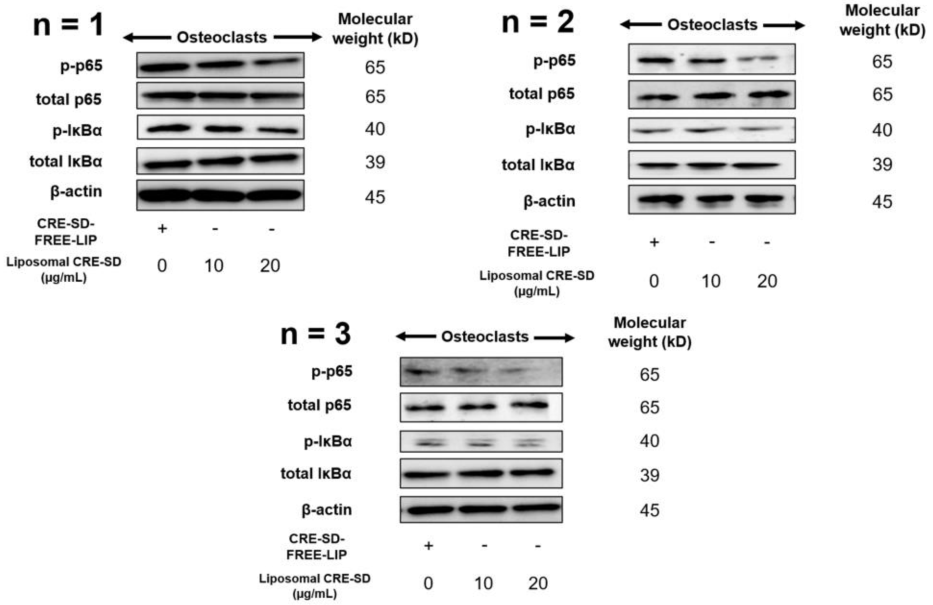

Figure S4. Triplicate images of blots analyzing 20 ng/mL RANKL-stimulated RAW 264.7 cells (osteoclasts), following the detection of the phosphorylation of p65 and IκBα. The 20 ng/mL RANKL-stimulated RAW 264.7 cells were treated with CRE-SD-FREE-LIP and liposomal CRE-SD (10 and 20 μg/mL).

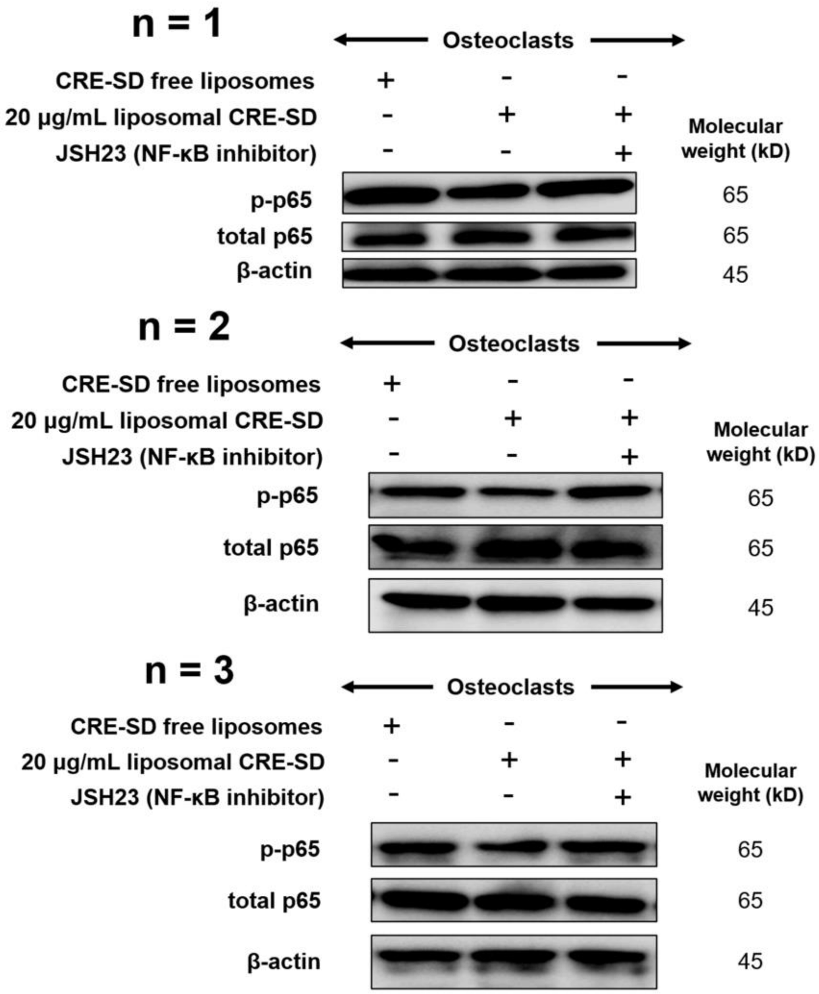

Figure S5. Triplicate images of blots of 20 ng/mL RANKL-stimulated RAW 264.7 cells (osteoclasts) for detecting the nuclear translocation and transcriptional activity of p-p65.

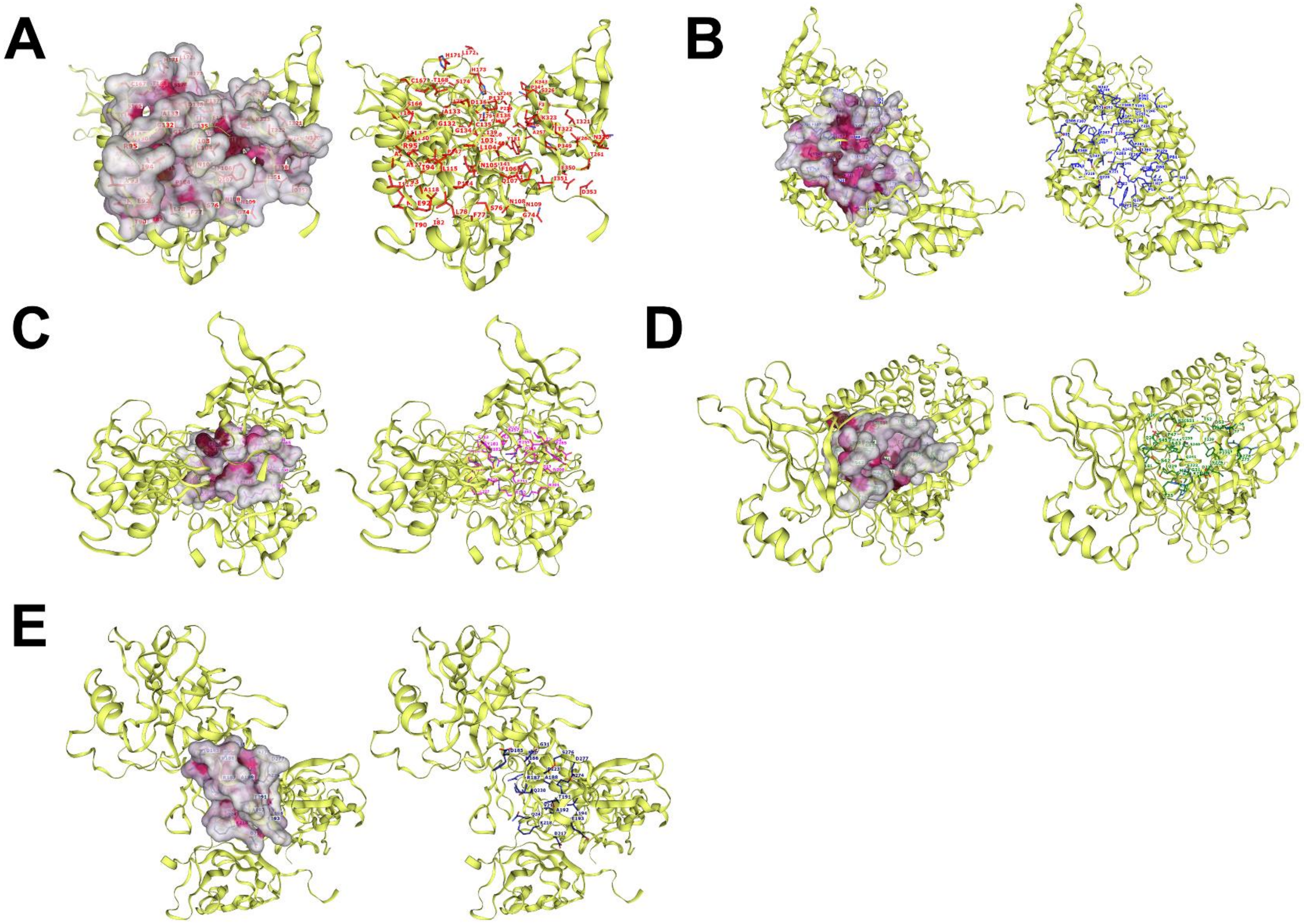

Figure S6: The 3D structures of five predicted binding pockets of the IκBα/p50/p65 protein complex derived from CurPocket. (A) Binding pocket 1. (B) Binding pocket 2. (C) Binding pocket 3. (D) Binding pocket 4. (E) Binding pocket 5.

Table S1: Possible contact residues of five predicted binding pockets of IκBα/p50/p65 protein complex derived from CurPocket

Binding pocket	Possible contact residues
1	Chain C<br>(SER326, LEU346, PRO344, THR256, PHE345, PRO324, ASN320, LYS343, THR261, VAL319, ASP353, ILE351, ALA257, VAL260, ILE321, LYS323, PRO349, GLU350, THR322)<br>Chain D<br>(GLY134, ASN105, CYS135, PHE103, GLY132, SER174, THR90, LEU78, TYR248, VAL93, HSD171, GLN111, ASN109, ARG140, LEU172, ILE175, LEU139, PHE77, THR179, PHE106, LEU130, LEU131, LEU115, GLY183, ASP136, ALA133, HSD173, ALA118, ASN108, MET91, ILE82, SER76, CYS167, ALA127, THR164, TYR181, THR169, THR168, SER166, PRO147, ILE126, LEU163, ILE94, GLU92, ARG95, ASP141, LEU104, GLN107, GLU138, GLY74, PRO137, ALA129, PRO114, ASN180, PRO214, THR113)
2	Chain A<br>(GLN29, LYS79, HSD181, HSD245, ARG158, GLU222, HSD83, ALA242, VAL248, ARG30, VAL244, ASP80, PRO182, ARG246, LYS221, VAL219, GLN247, GLN220, PHE184, PRO81, GLN241, LYS218)<br>Chain C<br>(ARG305, ASN247, VAL251, ASP271, LYS249, GLN306, PHE307)<br>Chain D<br>(THR247, TYR248, TYR289, ASP290, SER293, GLN212, MET279, SER283, GLU286, GLU287, GLU282, VAL246, GLY217, ARG245, SER288, TYR251, GLY250, PRO281, THR291, LEU280, GLN249, GLU292, TRP258)
3	Chain A<br>(ARG253, ARG201, GLU211, ASN200, PHE213)<br>Chain C<br>(THR256, ARG252, ARG255, ILE266, ALA257, GLU264, GLU265, TYR267, ASP254)<br>Chain D<br>(CYS215, TYR181, ILE192, ASN216, GLU213, ARG218, LEU223, HSD184, LEU227, ASP226, ASN182)
4	Chain A

Binding pocket	Possible contact residues
	(ARG273, GLN26, ASP53, GLN29, HSD181, SER276, ARG236, SER51, LYS28, GLU222, GLU225, VAL244, GLY44, ARG30, ARG33, ARG35, ARG278, GLY237, ARG50, LYS221, MET32, ILE224, PRO47, SER45, PHE239, SER42, ALA43, PRO27, THR52, SER238, GLU49, PRO275, ASP223, GLY31, SER240, GLN241)<br>Chain D<br>(ARG260, GLN266, GLY259, TRP258)
5	Chain A<br>(ASN186, ASP217, ARG274, ALA192, PHE184, ALA188, ARG187, VAL219, THR191, GLN247, LEU194, ASP185, SER276, ASP223, ARG30, GLN220, GLY31, ASP277, GLU193, LYS218)
